# Lightning Strike Protection: Current Challenges and Future Possibilities

**DOI:** 10.3390/ma16041743

**Published:** 2023-02-20

**Authors:** Markus Ostermann, Juergen Schodl, Peter A. Lieberzeit, Pierluigi Bilotto, Markus Valtiner

**Affiliations:** 1 CEST GmbH, Centre for Electrochemical Surface Technology, A-2700 Wiener Neustadt, Austria; 2 Institute of Physical Chemistry, University of Vienna, A-1090 Vienna, Austria; 3 Applied Interface Physics, Vienna University of Technology, A-1040 Vienna, Austria

**Keywords:** graphene, lightning strike, lightning strike protection, nanofiller, composite, composite production

## Abstract

An airplane is statistically struck by lightning every year. The need for lightweight aircraft to reduce the production of carbon dioxide has significantly reduced the presence of metals in favour of composites, resulting in lower lightning strike protection efficiency. In this perspective, we critically review the state of technologies in lightning strike protection solutions based on carbon materials, graphene, and MXenes. Furthermore, we comment on possible future research directions in the field.

## 1. Introduction

### 1.1. The Problem of Lightning Strike Protection

The strike of global climate crisis is driving research on novel solutions to reduce the impact of human industries on the planet. For instance, the CORSIA (Carbon Offsetting and Reduction Scheme for International Aviation) program sets a scheme towards a sustainable aeronautical industry increasing the demand for novel efficient composite structures [1] with the goal to reduce the production of carbon dioxide [2].

**Figure 1:**
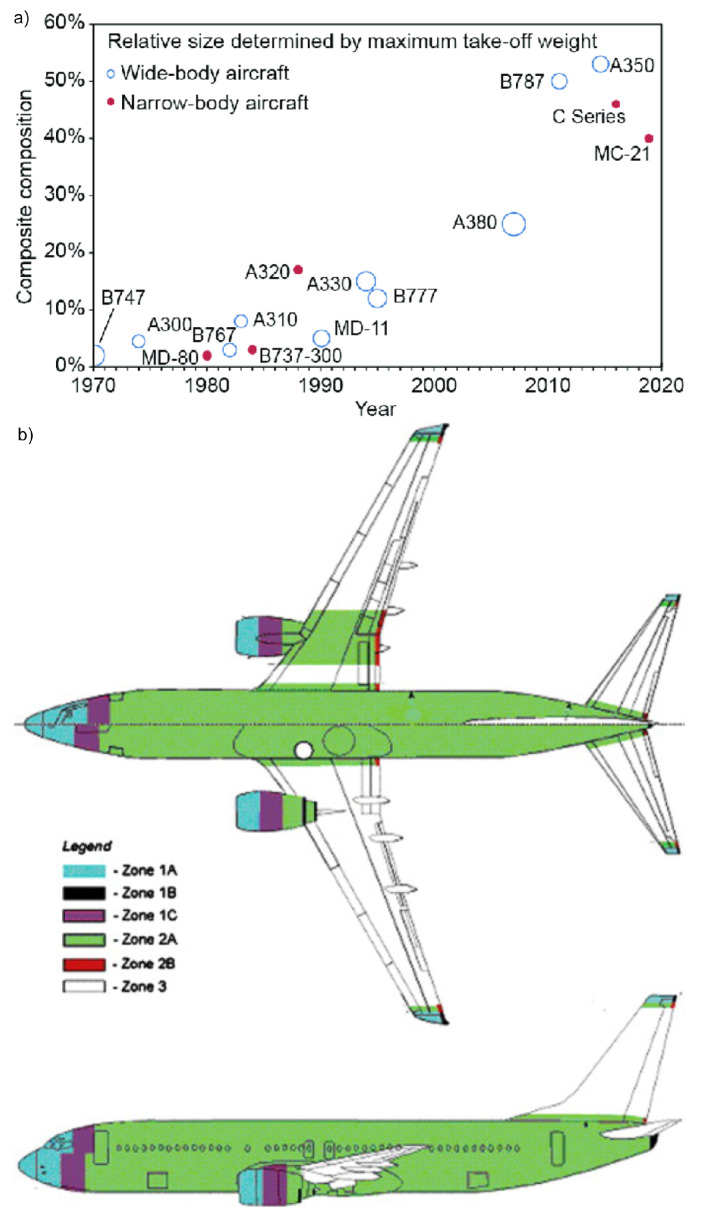
(**a**) Amount of composite material used in commercial aircraft since 1970 [3]; (**b**) lightning strike zones of a commercial aircraft according to SAE ARP 5414. (Reprinted from Feraboli et al. (2009) [7] under permission from Elsevier).

In the last 50 years, the percentage of composite materials in structural parts of modern passenger aircraft increased from 5 to 50 % with a substantial optimization of the aircraft weight (see Figure 1a) [3,4]. Lightning strikes are a rather frequent event in aeronautics (every commercial plane is statistically hit once per year), consequently, every aircraft needs to express a proper lightning strike protection (LSP) to avoid serious damage as regulated by FAA AC25-21 [5,6]. In detail, SAE ARP 5414 defines lightning strike zones (see Figure 1b), whereby Zone 1 is prone to initial lightning attachment and first return strokes and Zone 2 (the majority of the airframe) experiences swept strokes or re-strikes [7]. A strategy of simply substituting metal (alumina) parts with polymer composite materials cannot work in the aeronautic industry because of the consequent reduced conductivity. The resulting low LSP performance increases the risk of mechanical damage in the structure, which can lead to on-cruise accidents in the worst case scenario.

**Figure 2:**
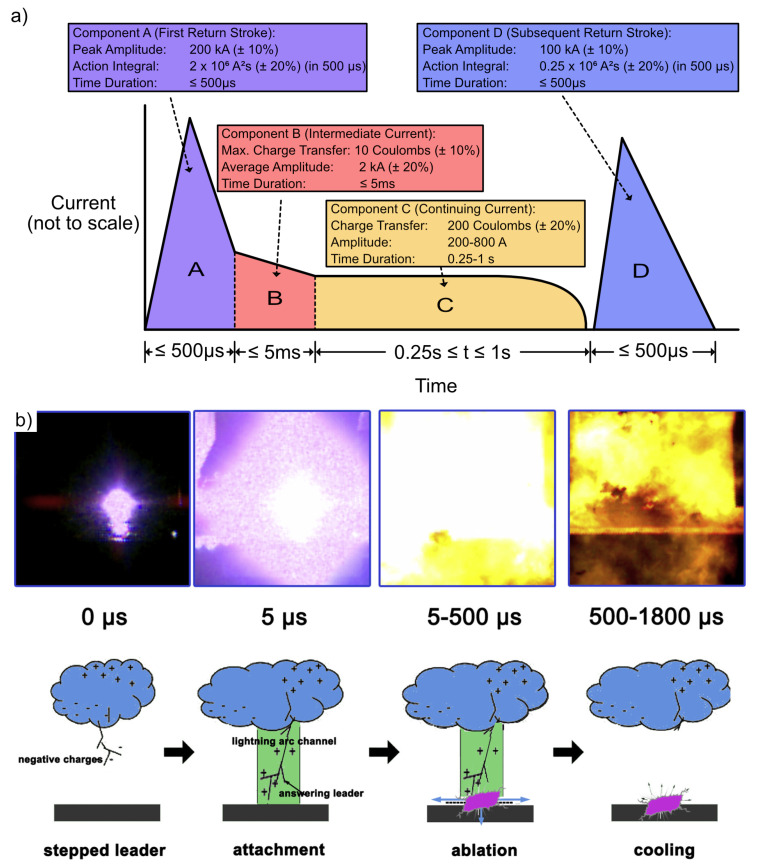
(**a**) Typical simulated lightning current waveforms according to SAE ARP 5412 (adapted from Feraboli et al. (2009) [7] under permission from Elsevier); (**b**) sequence of a lightning strike recorded with a high-speed camera (reprinted from Wang et al. (2020) [8] under permission from Elsevier).

Thus, in recent decades efforts have been made to clarify, on the one hand, how lightning strikes and the damage evolves, and on the other hand, how to optimize coatings able to sustain such a short transfer of electrical current along the aircraft surface. To test the LSP capability of aeronautical structures, SAE ARP 5412 defines typical lightning flash current waveforms (see Figure 2a). Component A represents a first return strike, Components B and C represent long duration currents following return strokes and re-strikes, and component D represents a subsequent stroke. Lightning Zone 1 is required to sustain waveforms A–C, whereas Zone 2 needs to sustain only components B–D. To meet manufacturer requirements, airframes need to sustain 70% of their design limit load (DLL) after a high-energy strike (50–200 kA), 100% of the DLL after an intermediate energy strike (30–50 kA; visible damage needing repair), and 150% of the DLL after a low-energy strike (10–30 kA; no or barely visible damage). Lightning-induced damage was discussed in 2009 by Feraboli et al. by analysing the performance of carbonfibre-reinforced polymer (CFRP) upon an intermediate energy strike (30–50 kA) [7]. In the work, a major decrease in mechanical properties was discussed, which highlighted the need for better understanding the entire process. Wang et al. proposed in 2020 an interpretation on how damage on CFRP is formed when lightning strikes (see Figure 2b) [8]. The initial attachment of the strike takes about 5 µs until breakdown of the electric field of the air. This attachment to the test panel induces rapid temperature increase due to Joule’s heating. After the attachment, temperature starts distributing along the fibre direction accompanied by sample ablation. With the conductive fibres acting as an electrical channel, arcing between fibres increases matrix decomposition. Initial high temperature zones at the attachment point spread across the top layers. About 500 µs after the strike, the sample steadily cools down, whereby the heat is also transferred to underlying layers causing matrix decomposition. Cooling under the decomposition temperature takes at least 3 s. The following damages of the CFRP include matrix vaporization, intraply cracks, delamination, ply-lift, and fibre breakage mainly related to the arc flow along the fibre direction and thereby induced Joule’s heating of the material [8,9]. The dynamic proposed here is convincing and further highlights the need for methods that can improve LSP, for instance by adding conductive structures to redirect currents induced by lightning in the composite [6].

Still, this task is challenging because the coating needs to express multifunctionalities such as light weight and reduced thickness, resistance to erosion, adhesion to the composite structure, great mechanical stability to avoid crack propagation, and stability in a large range of temperature that would allow an aircraft to lift off from an airport with 40 °C, operate at temperatures down to −40 °C in-flight, and land in one with temperatures of 0 °C.

Herein, we report the current solutions proposed in terms of LSP. Then, we critically discuss the possible future trends in this field and materials that could make a breakthrough in this research field.

### 1.2. Metallic Solutions for LSP

Preferential solutions for effective LSP are metallic meshes (e.g., copper, alumina) integrated in the outer layers of the composite structure via impregnation with adhesives or sandwiching between two adhesive films. Providing reasonable conductivity, the copper meshes channel electrical currents and reduce thermal stress on the composite. By tuning the mesh thickness and open hole area, performance of the metallic mesh can be adjusted to the LSP requirements. If correctly applied, metallic meshes can limit the damage to a small area (few 100 mm^2^) of the surface without reasonable depth penetration. Commercial LSP systems use copper meshes with a thickness of about 0.10–0.20 mm [10,11,12]. Clearly, the addition of metallic meshes adds a significant amount of weight (150–200 g/m^2^) to the structure, dampening the positive weight-saving effect of composite usage. Moreover, metallic meshes (especially alumina meshes) are prone to corrosion deteriorating their performance within the composite system. Therefore, careful design avoiding galvanic corrosion (e.g., with an additional glass-fibre separation layer) or corrosion-resistive coating of the mesh is necessary. Further, delamination problems can be tackled with adhesion-enhancing coatings on the copper meshes [12,13].

Alternatively to metallic meshes, the integration of metallic fibres into the fibre ply or the application of metallic coatings onto the fibres can provide a similar protection mechanism, although corrosion and fibre/matrix adhesion challenges remain to be solved [14]. Metallic paints (most commonly silver) applied by various spraying methods are also a valid alternative to metallic meshes, but they are expensive and increase the overall coating layer thickness. Further, they are often not suitable to be considered a standalone LSP system [11,12].

## 2. Carbon-Based LSP Solutions

### 2.1. Carbon Black and Carbon Nanotubes for Lightning Strike Protection

In recent decades, conductive carbon materials emerged as an alternative to metallic systems offering good conductivity and easy processing for lightweight composites. The use of carbon nanofillers offers the possibility to introduce lightning strike protection into the matrix structure and is therefore favourable in saving process steps during production [12]. Carbon nanotubes (CNT) are rolled carbon sheets forming tubes exhibiting good electrical conductivity and high aspect ratio, wherefore they are a viable carbon-based candidate for LSP application [15]. Mechanical mixing of CNTs into an epoxy matrix expresses an electrical conductivity of up to 6 × $10^{-3}$ S/m at 0.75 w% CNT concentration [16]. Using compression moulding, higher CNT concentrations up to 80 w% can be prepared showing significantly higher conductivity (up to 838 S/m) [17]. Similar concentrated CNT films incorporated in a composite structure are able to significantly reduce lightning-induced damage by 77.6% and 68.0% in area and depth, respectively. Thus, these solutions are comparable to a silver paint with 50% less weight addition to the system [18]. To improve LSP performance, combinations of CNTs and carbon black (CB) with up to 0.75 w% filler can be considered and compared to the addition of CNTs/CB solely. In the mixed case, the lightning (100 kA)-induced damage significantly decreased, with a retention of flexural modulus up to 95.0% (0.125% CB + 0.125% CNT) as well as flexural strength up to 87.3% (0.75 w% CNT), proving the synergistic effects [19]. With CNT- and CB-based solutions being promising, but still insufficient for a standalone LSP system, the spotlight was also put on graphene-based solutions to reach requirements.

### 2.2. Graphene-Based Solutions for Lightning Strike Protection

The need for higher conductivity in composites drives the application of two-dimensional materials (2DM) in aeronautical coating research. Since its discovery in 2004 [20], graphene has been highly investigated due to its high conductivity, light weight, good tribological properties, hydrophobicity, and high surface area [21]. Due to these properties, it is of high interest for the aeronautical industry in various fields of application such as de-icing [22], flame inhibition [23], water uptake, and corrosion protection [24,25]. With an electrical conductivity of about 1.46 ×$10^{6}$ S/m [26], a thermal conductivity of 4.84 ×$10^{3}$ W/mK [27], and a density of 2.267 g/cm^3^, single-layer graphene sheets appear an ideal solution for LSP. Although these properties are promising, technical realisation of graphene-based LSP systems appears challenging.

#### 2.2.1. Mechanical Mixing

To form a conductive network within the composite, implementation of the graphene filler is the most important step. A simple form of implementation is mixing it into a polymer matrix by high-shear mixing [28,29], calendaring [22,30], or ultrasonication [24]. The graphene filler is uniformly dispersed within the matrix. When excelling the percolation threshold, a conductive network of the graphene sheets is established and the resulting composite structure shows reasonable conductivity. Graphene-doped composites with electrical conductivity up to 100 S/m have been demonstrated via mechanical mixing methods [22,29,30,31]. These conductive capabilities are suitable for applications such as electrothermal heating, but the full potential of the graphene sheets is not harnessed with mechanical implementation due to problems such as aggregation or polymer films between particle interfaces. Therefore, composite conductivity appears to be too low to withstand lightning currents up to 200 kA, making alternative implementation processes necessary for LSP.

#### 2.2.2. Vacuum-Assisted Implementation Techniques

To use the full potential of graphene sheets, confined systems are proposed for LSP concentrating graphene in thin layers within the composite.

A simple approach to produce concentrated graphene films represents vacuum filtration of previously dispersed graphene sheets. Following filtration, a compression step increases film stability and uniformity producing standalone films with thicknesses of about 25 µm to 150 µm. Electrical conductivity of these compacted graphene sheets are in the range of 1.32 ×$10^{4}$–1.76 ×$10^{5}$ S/m [32]. By incorporating the films into the composite structure, the lightning-induced damage area (100 kA strike) was decreased by 94% compared to the reference sample. Still, such a performance is accompanied by mechanical instability due to interlayer delaminations formed in the composite [33]. The latter highlights a major problem of film-based LSP solutions as the matrix impregnation of the film and the adhesion between matrix–film is insufficient to generate a mechanically stable system. To overcome delamination problems, techniques such as 3D stitching are proposed to increase stability in the z-direction and maintain tensile strength as well as conductivity, but the strength is strongly dependent on the stitching parameters and increases the complexity of the composite.

**Figure 3:**
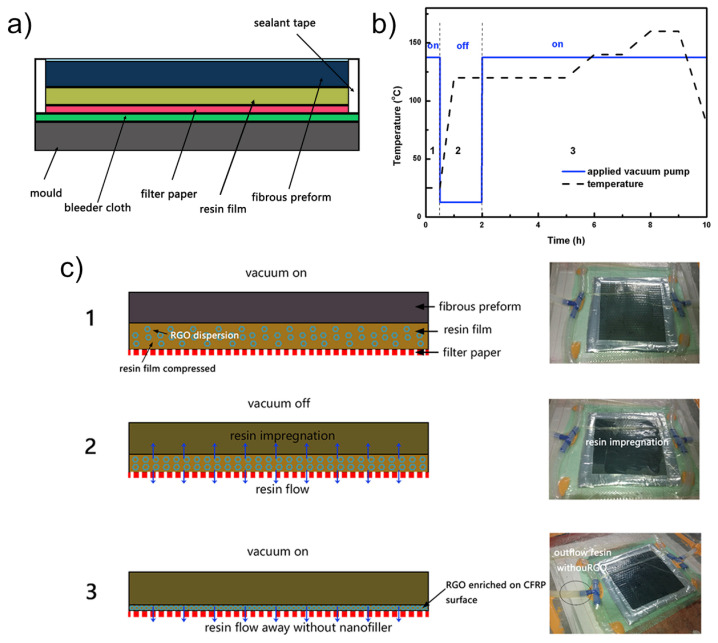
Resin film infusion (RFI): (**a**) scheme of the preform fabrication, (**b**) temperature and vacuum protocols used for moulding the CFRP, and (**c**) schematic of the RFI process to accumulate RGO on the CFRP surface and images of the RFI process. (Reprinted from Wang et al. (2018) [34] under permission of Elsevier).

Alternatively, production of concentrated graphene layers during vacuum bagging is achieved via resin film infusion (RFI) [34]. By mixing reduced graphene oxide (rGO) into an epoxy blend, a polymer/rGO slurry was produced. This slurry was placed on top of filter paper in a mould and covered with a fibrous preform (see Figure 3a). Via a temperature- and vacuum-assisted process (see Figure 3b), the resin impregnates the fibrous preform, whereby rGO is concentrated between the preform and the filter paper (see Figure 3c). The technique produces concentrated rGO layers with thicknesses of about 40 µm incorporating up to 9 g nanofillers/m^2^. Further increase in rGO concentration is not possible due to flakes blocking the resin flow through the filter paper at higher amounts. Surpassing the percolation threshold at 2 g/m^2^, the layers sheet resistance is less than 0.55 Ω/sq (equal to a conductivity of 3.7 ×$10^{5}$ S/m, respectively, to the sheet thickness), appearing superior to vacuum-filtration-based film production. Lightning strike tests (40 kA) showed the capability of the graphene layer to divert the current and significantly reduce Joule’s heating. This results in a decrease in the lightning-induced damage area by a factor 4. Ultimately, the composite retained 77% of its flexural strength, while the unprotected composite only achieved 34% of its initial strength.

Compared to ex-situ vacuum-filtration-based film production, in-situ RFI produces thinner concentrated layers with superior conductivity and avoids delamination problems by ensuring impregnation of the rGO layer during composite production. On the downside, the technique is limited to loadings up to 9 g/m^2^ hindering performance improvements with higher concentrations. Deteriorating flow dynamics within fibre impregnation, potentially leading to irregularities in matrix distribution, could also be a problematic factor during industrial processing. Nonetheless, vacuum-assisted techniques appear superior to mechanical mixing in terms of LSP application.

#### 2.2.3. Alternative Film Production

Beside vacuum-assisted film production, production in combination with additives can be used for producing conductive graphene films. Commonly, polymers, such as polyvinyl alcohol (PVA) [35], polyaniline (PANI) [36], polyvinylpyrrolidine (PVP) [36], or epoxy resin [28], are viable options to stabilize graphene films prior to implementation into a composite structure. Using PVA, evaporation casting produces films with conductivity up to 6.6 ×$10^{3}$ S/m, suitable for the study’s intended electrothermal heating element, but insufficient for LSP [35]. Similarly, a combination of graphene platelets with the conductive PANI and the stabilizing PVP produces films with up to 25 S/m [36]. Graphene/PANI/PVP incorporated in a CFRP sandwich structures via hot pressing, shows minimal damage at an overall graphene loading of 11 w%, but subsequent mechanical tests outline a major problem of these sandwich structures: due to the minimal damage the mechanical strength retention is significantly higher (70% compared to 50% of the reference), but the addition of the graphene films decreases the overall mechanical strength of the composite structure significantly.

**Figure 4:**
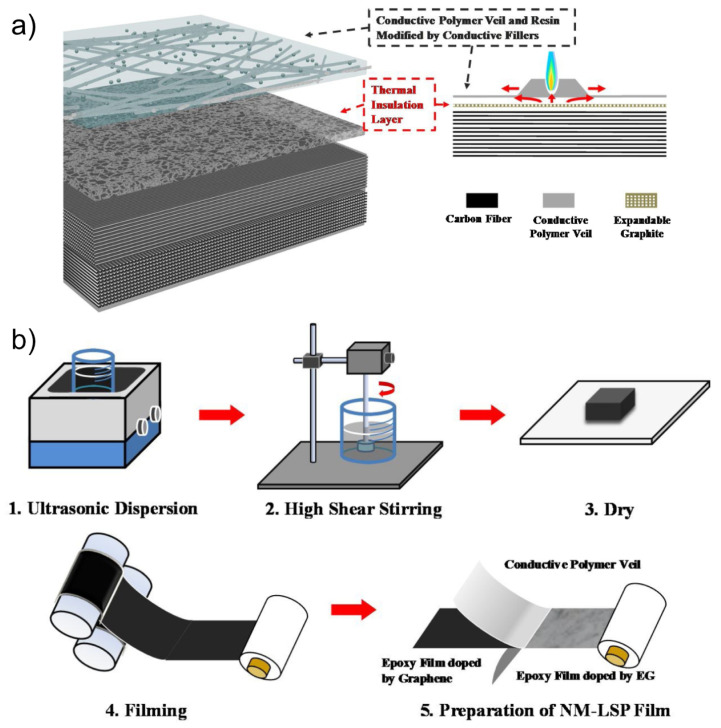
Two-layer NM-LSP film: (**a**) proposed two-layer design of a conductive and a thermally insulating layer based on a polymer veil, graphene, and expanded graphite; (**b**) production of this NM-LSP film by heat rolling (reprinted from Zhao et al. (2020) [28] under permission of Elsevier).

Nonetheless, addition of polymers to graphene films deteriorates the electrical conductivity, being about two magnitudes lower than the conductivity of pure graphene films. Conductive enhancement of polymer/graphene films can be achieved via incorporation of metallised polymer veils [28]. The silver-plated polymer veil shows sheet resistances down to 0.07 Ω/sq. Graphene (0.25 w%) incorporated in an epoxy matrix via ultrasonication and high-shear mixing, impregnated in the polymer veil, results in composite resistances down to 3.7 Ω/sq. This LSP film is able to reduce the damage area by 71 % (100 kA strike). Similar to other polymer/graphene films, the resistance is still too high to fully avoid damage caused by lightning-induced Joule’s heating. Therefore, improvement via a two-layer design incorporating an electrically conductive and a thermally insulating layer to achieve sufficient LSP protection (see Figure 4a) was employed. The secondary layer was produced by doping an epoxy composite with expandable graphite and compressing it together with the graphene/epoxy matrix and the silver-modified polymer veil (see Figure 4b). The resulting two-layer system matched the LSP performance of a commercial copper mesh by diverting the current within the conductive layer and protecting underlying structures from heat-induced decomposition.

A summary of the mentioned conductive graphene-based composites is shown in Table 1.

#### 2.2.4. Comparison between Graphene-Based Solutions and Copper Mesh

To achieve conductivity comparable to currently used copper mesh, implementation techniques producing confined layers of graphene are necessary. Vacuum-assisted infusion [34] or film production [32,33] thereby show the most promising results to exchange copper in lightning Zone 2 possibly withstanding subsequent strokes up to 100 kA. Nonetheless, to adapt the proposed systems to industrial application in structural airframe parts there are many challenges related to the upscalability.

For instance, large scale films are often fragile due to a lack of stabilizers. Adding stabilizing polymers is not an ideal solution as they will hamper the conductivity of the film [36]. Additionally, delamination problems on the film–composite interface [33] induce significant reduction in mechanical stability [36]. A possible route to avoid delaminations is impregnation of the films into the polymer matrix, although dense graphene films are difficult to impregnate while keeping high conductivity. Another relevant issue related to multilayer preparation is the adhesion of the graphene layer and ultimately of the entire coating. If the graphene-based LSP layer is the outermost layer, additionally to the adhesion to the structural composite, adhesion to functional coatings should also be ensured. This requires adaptations of coating formulations to new LSP systems, making a direct substitution of current metallic LSP systems difficult.

Another element that goes against the substitution of copper mesh with graphene-based materials is the production cost of high-quality graphene. Prices are about 94 USD/m^2^ for an aeronautical LSP copper mesh [38], whereas rGO material for realisation of the system proposed by Wang et al. [34] costs about 200–900 USD/m^2^ [39]. Still, if the performance of the rGO system could be comparable to the copper mesh, the achieved weight savings of up to 99% would have a higher economic impact than the material costs. In addition, the production cost of graphene and its derivatives could be optimized potentially down to ≈1 USD/g for rGO, facilitating the transition from copper mesh solutions.

## 3. Enhancement of Graphene LSP Properties

The mentioned challenges of performance enhancement, delamination, and improvement of integration and material costs need to be solved to achieve industrialisation of graphene-based LSP systems. This goal can be reached by acting directly on the graphene preparation or by combining it with metals such as silver nanoparticles or other 2DM.

The aspect ratio is a crucial factor on the conductive performance of graphene nanofillers in composites. Higher aspect ratios improve the performance, significantly lowering the percolation threshold and reducing contact resistance between particles [40,41]. Possibilities to enhance the aspect ratio in exfoliation processes include, for example, the minimisation of shear forces [42] or the exfoliation of MWCNTs to produce high-aspect graphene nanoribbons [43]. In disturbing the aromatic structure and therefore the electrical conductivity, in-plane defects of the material need to be minimized. By choosing solvents with suitable surface energies, such as N-methyl-pyrrolidone (NMP) and dimethylformamide (DMF), liquid exfoliation producing defect-free graphene was achieved. Transferring the graphene flakes to solvents such as acetone and ethanol via ultrasonication makes them suitable for practical application in composite systems and at the industrial scale [44,45].

### 3.1. Silver Doped Graphene Composites for LSP

In principle, single-layer graphene sheets would be the most suitable option concerning LSP, but the production of defect-free single-layer graphene via CVD is costly, wherefore exfoliation of graphite is a preferred route for industrial application. Graphite is intercalated in aqueous solution by various agents (e.g., sulfate ions, alkali ions, ⋯), leading to exfoliation. This often produces defects within the aromatic structures, such as oxidic groups or crystal defects deteriorating the conductivity of the flakes.

**Figure 5:**
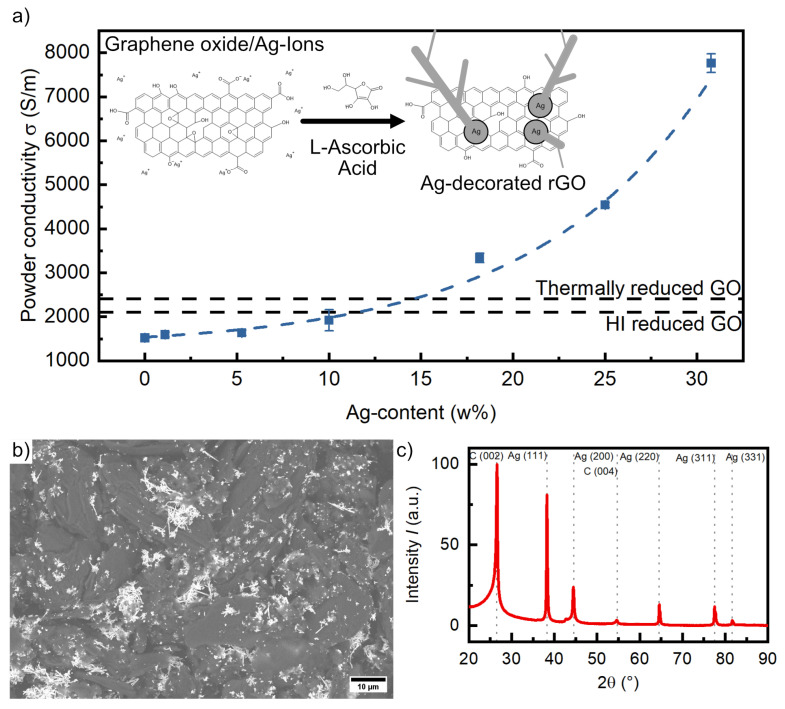
Preliminary results of the Ag^+^ and graphene oxide co-reduction by L-Ascorbic acid: (**a**) powder conductivity of Ag/rGO powders against the Ag content (Insert: reaction scheme); (**b**) SEM micrograph showing dendritic Ag particles deposited on rGO flakes; (**c**) X-ray diffractogram of a rGO/Ag powder showing no sign of oxidation/impurities.

To enhance the graphene oxide conductivity, reduction of the oxidic groups is necessary to restore the aromatic network. Various possible reduction routes such as chemical [46,47], thermal [48], solvothermal [49], and photoinduced [50] reduction are reported in the literature, with the first two being the most prominent. For example, powder conductivity of HI-reduced GO was measured at 2.11 ×$10^{3}$ S/m, whereas thermally reduced GO achieved conductivity up to 2.41 ×$10^{3}$ S/m (see Figure 5a) [48]. L-Ascorbic acid is a green and cheap alternative reductant showing similar capability to more toxic agents such as hydrazine or hydroiodic acid, producing reduced graphene oxide (rGO) powders with conductivity of 1.52 ×$10^{3}$ S/m. The reduction process furthermore offers the possibility of co-reducing metal ions to form metal particles on the rGO surface. This technique was prominently investigated aiming for antibacterial properties of Ag/rGO and Cu/rGO powders [51,52]. By adding Ag^+^-ions prior to the reduction process, graphene oxide and Ag^+^-ions are simultaneously reduced to rGO and metallic silver by L-Ascorbic acid (see Figure 5a Insert). The powder conductivity (measurement setup adapted from Celzard et al. [53]) increases exponentially with the Ag content (see Figure 5a), ultimately reaching a conductivity of 7.76 ×$10^{3}$ S/m at 30 w% Ag addition. This corresponds to an increase of about a factor of 5 compared to pure rGO. SEM investigations (see Figure 5b) confirm the presence of dendritic Ag particles uniformly distributed on the rGO flakes. The increment in conductivity is related to the Ag particles interconnecting rGO flakes, reducing contact resistance between particles. A XRD diffractogram (see Figure 5c) confirms the purity of the Ag-modified rGO powder. Within the diffractogram, only reflexes related to metallic silver (crystallite size ≈ 35.2 nm) and rGO are observed. With silver oxide reflexes not being present in the diffractogram, the risk of silver oxidation hampering the conductivity is excluded. This stabilizing effect on the Ag particles is related to rGO acting as a cathodic protection, ensuring reliable electrical performance [54]. Finally, composite layers resulting from this approach take advantage of both the high electrical conductivity of Ag and the cheap reduced graphene oxide, providing a basic electrical and thermal conductivity as well as protecting Ag particles from oxidation.

### 3.2. Graphene/CNT Composites

Another approach to reach suitable conductivity for LSP could be graphene/CNT hybrid systems. By co-dispersing CNTs and graphene flakes followed by a vacuum-filtration-assisted process, Tang et al. [55] were able to produce a graphene/CNT hybrid paper. At an approximate thickness of 200 µm, the paper, with a conductivity of 7.7 ×$10^{3}$ S/m, is reported at a CNT:graphene ratio of 3:1. This outperforms both the pure CNT paper (3.3 ×$10^{3}$ S/m) and graphene paper (6.7 ×$10^{3}$ S/m), proving a synergetic effect of the carbon-based materials. As discussed with silver, the effect relies on the conductive networks being improved because of interconnections decreasing the contact resistance of the system, and on graphene flakes being able to maintain high aspect ratios of the CNTs. However, the conductivity of the hybrid film is still not enough and would need improvements to be considered for LSP application.

### 3.3. MXenes for LSP

**Figure 6:**
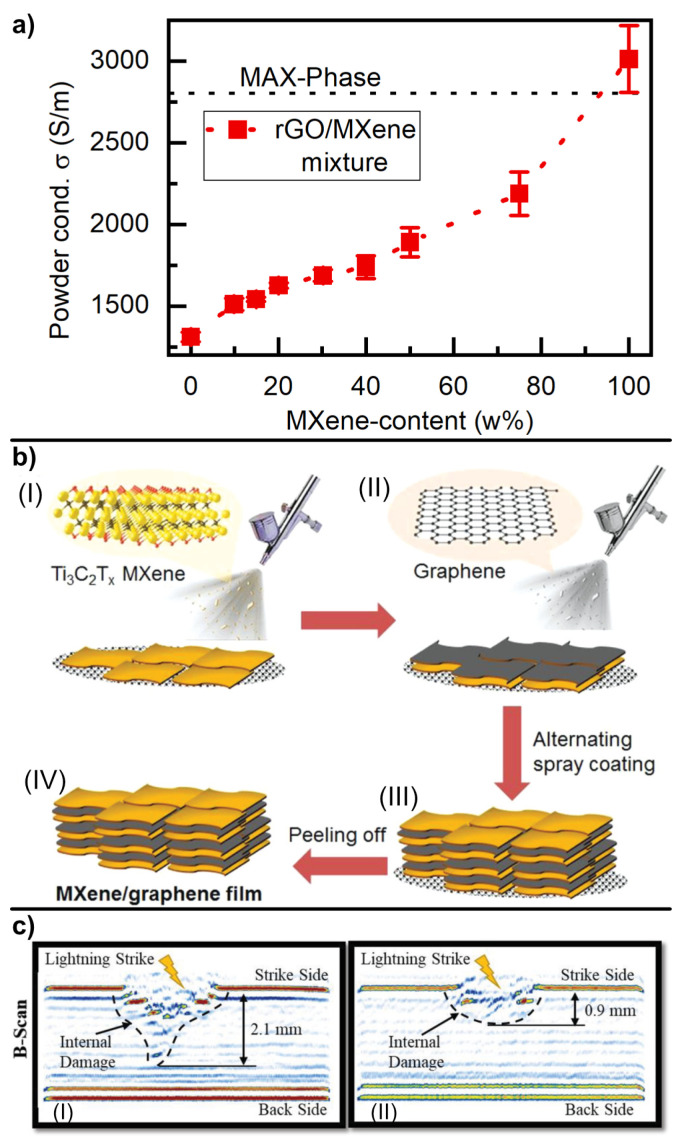
(**a**) Powder conductitivity of rGO/MXene mixtures against the MXene content; (**b**) application of rGO/MXene multilayered film system by alternated spray-coating: (I) spraying of Ti_3_C_2_T_x_ MXene nanosheets, (II) spraying of rGO nanosheets, (III) production of heterogenic structure by alternate spray coating, (IV) Peel-off of the heterogenic MXene/rGO film electrode (reprinted from Zhao et al. (2019) [61] under permission from Wiley-VCH); (**c**) penetration depth evaluation following a lightning strike of an unprotected CFRP (left) and an MXene LSP-system-protected CFRP (right) (reprinted from Kumar et al. (2021) [62] under permission from Wiley-VCH).

An emerging field of 2DM is MXenes (early transition metal carbides and nitrides) with their currently most prominent material Ti_3_C_2_T_x_ [56]. Recent material-synthesis-related studies [57] were able to improve MXene conductivity to 2.4 ×$10^{6}$ S/m, being superior to graphene sheets (1.46 ×$10^{6}$ S/m [26]) and exhibiting breakdown currents comparable to graphene (1.2 ×$10^{8}$ V/cm^2^) [58]. In addition, MXenes can express a variety of surface terminations, which can be modified to improve adhesion to the matrix, but they carry the drawback of a higher density (4.21 g/cm^3^ [59]) compared to graphene. It is worth noting that related to the material density, the conductivity of MXenes (5.7 ×$10^{5}$ S/m per g/cm${}^{3}$) and graphene (6.4 ×$10^{5}$ S/m per g/cm${}^{3}$) is very similar. Consequently, combination of the positive effects of MXenes and graphene is of high interest. Preliminary conductivity measurements of rGO/MXene powder mixtures (See Figure 6a) show a significant increase related to the addition of MXenes. Thus, with both materials providing unique electrical characteristics, MXene termination could act as a bonding site to the composite, generating adhesion, whereas the addition of graphene lowers the overall density of the material as well as the material costs.

Additionally, the combination of rGO and MXenes offers the possibility of layer self-assembly to generate an oriented network, i.e., heterostructures [60]. It has also be demonstrated that the latter can be produced on a large scale by alternating spray-coating of MXenes and rGO (see Figure 6b) [61].

Direct application of MXenes in lightning strike tests comes as follow. MXene-doped thermosetting coatings with a conductivity of about 100 S/m showed reduction in lightning-induced surface damage of a CFRP panel by 67% (see Figure 6c), while retaining 86% and 78% residual flexural strength and modulus of the composite, respectively [62]. An alternative strategy toward LSP and light weight is a minimization of metals in composites, which are reinforced by 2DM to secure the conductivity. For instance, a combination of MXenes and 10 w% copper nanowires has been recently tested to provide lightning strike protection for a current of 100 kA [63].

## 4. Conclusions and Outlooks

The demand for lightweight aircraft to reduce the production of carbon dioxide reveals the need for advanced lightning strike protection (LSP) that does not rely on metal materials. Two-dimensional-material-based LSP systems show promising results to substitute currently used copper mesh in LSP Zone 2. Specifically, the utilization of graphene, MXenes, or a combination of both, has provided inspiring results to address LSP. Still, there are many aspects to keep in consideration while researching 2DM-LSP.

First, the fusion of 2DM films and composites is not optimized to the prototype or higher technological level of readiness yet. This implies fundamental investigation on the functionalisation of 2DM to improve the contact points between 2DM flakes dispersed in a composite. Surface analysis will be crucial in this effort, comparing techniques to verify active functional groups and quantify elements at the first atomic layer (e.g., XPS, XRD, AES, LEIS, ⋯). Moreover, further improvement in 2DM research will need to pay attention to the environmental impact of the chemistry in use. In this sense, green chemicals are essential for industries to comply with the goals set in the European Green Deal, as discussed in our previous work [48].

Second, instead of pursuing a no-metal strategy, it could be beneficial to approach the challenge as a minimization problem. For instance, the presence of copper in a mesh could be reduced to particle distribution in 2DM composites. As shown in Figure 5, the presence of silver particles in rGO can effectively increase the electrical conductivity. Such a system is not optimized yet, resulting in a loss of conductivity due to poor control on the interconnection. A possible future research direction in this field could deal with upscalable green mechanisms able to maximise interlocking of flakes and metal nanoparticle distribution according to a cost–benefit analysis of the quantities in play (i.e., weight percent of metals nanoparticles with respect to 2DM).

Third, MXenes are undoubtedly one of the most promising 2DMs in terms of conductivity and versatility [56]. The most studied, and better understood, is titanium carbide. It is worth noting that starting from October 2022, titanium has made its appearance in the critical raw materials list [64]. Considerations on raw materials must be taken into account for future research in accordance with the sustainability and development goals (SDG) proposed by the United Nation (specifically, SDG 12 and 13). In the specific case of MXenes, the attention to this topic could be a boost toward investigating highly conductive MXenes not based on titanium. This is surely an exciting field direction given the wide possibilities that MXenes have in terms of experimental discovery of new compounds that are only theoretically predicted [65].

Fourth, higher conductivity might not necessarily mean a better LSP performance. As discussed in the work of Zhao et al. [28], despite a lower conductivity, a multi layer system is able to show superior LSP than more conductive systems. Thus, future investigation on multilayer architectures with novel materials expressing electrical and thermal synergistic properties could provide a breakthrough in the field.

In conclusion, although graphene-based LSP systems could reduce weight up to two order of magnitude to the currently used copper meshes, further investigation is essential to obtain a light composite material able to sustain a lightning strike. Thus, 2DMs are probably the key area that as scientists we must exploit in order to unlock this technology and fly on safe and environmentally friendly aeroplanes. 

## Figures and Tables

**Table 1:** Conductivity of various graphene-based composites compared to a copper mesh.

	Production Mechanism	Conductivity [S/m]	Reference
Reference	Copper mesh for LSP	2.2 × $10^{6}$–1.2 × $10^{7}$ (depending on the direction)	Guo et al. [37]
1	Mechanical mixing into epoxy matrix	>1.0 × $10^{2}$	Raji et al. [31]
2	Mechanical mixing into epoxy matrix	4 × $10^{-3}$	Prolongo et al. [30]
3	Mechanical mixing into epoxy matrix	3.8 × $10^{-3}$–8.4 × $10^{-3}$	Redondo et al. [22]
4	Mechanical mixing into epoxy matrix	6.0 × $10^{-1}$	Li et al. [29]
5	Vacuum-filtration assisted film production of graphene film	1.3 × $10^{4}$–1.8 × $10^{5}$	Zhang et al. [32]
6	Vacuum-filtration assisted film production of graphene film	1 × $10^{4}$–1.8 × $10^{5}$	Kumar et al. [33]
7	Resin film infusion (RFI)	3.7 × $10^{5}$	Wang et al. [34]
8	Dispersion and evaporation casting of a graphene/PVA film	6.6 × $10^{3}$	Vertuccio et al. [35]
9	Production of graphene/PANI/PVP film	2.5 × $10^{1}$	Lamichhane et al. [36]
10	Comb. of graphene-enhanced matrix and metallised polymer veil	2.7 × $10^{1}$ (Matrix); 1.4 × $10^{3}$ (Fiber)	Zhao et al. [28]
11	Spray-coating of rGO particles on glass-fibre composite	5.0 × $10^{0}$–3.3 × $10^{2}$	This work

## Data Availability

The data presented in this study are available upon request from the corresponding authors.
